# Temporally and spatially partitioned behaviours of spinner dolphins: implications for resilience to human disturbance

**DOI:** 10.1098/rsos.160626

**Published:** 2017-01-11

**Authors:** Julian A. Tyne, David W. Johnston, Fredrik Christiansen, Lars Bejder

**Affiliations:** 1 Murdoch University Cetacean Research Unit, School of Veterinary and Life Sciences, Murdoch University, South Street, Murdoch, Western Australia, Australia; 2 Division of Marine Science and Conservation, Nicholas School of the Environment, Duke University Marine Laboratory, 135 Duke Marine Laboratory Road, Beaufort, NC, USA

**Keywords:** behavioural strategies, human activities, conservation measures, Hawaii

## Abstract

Selective forces shape the evolution of wildlife behavioural strategies and influence the spatial and temporal partitioning of behavioural activities to maximize individual fitness. Globally, wildlife is increasingly exposed to human activities which may affect their behavioural activities. The ability of wildlife to compensate for the effects of human activities may have implications for their resilience to disturbance. Resilience theory suggests that behavioural systems which are constrained in their repertoires are less resilient to disturbance than flexible systems. Using behavioural time-series data, we show that spinner dolphins (*Stenella longirostris*) spatially and temporally partition their behavioural activities on a daily basis. Specifically, spinner dolphins were never observed foraging during daytime, where resting was the predominant activity. Travelling and socializing probabilities were higher in early mornings and late afternoons when dolphins were returning from or preparing for nocturnal feeding trips, respectively. The constrained nature of spinner dolphin behaviours suggests they are less resilient to human disturbance than other cetaceans. These dolphins experience the highest exposure rates to human activities ever reported for any cetaceans. Over the last 30 years human activities have increased significantly in Hawaii, but the spinner dolphins still inhabit these bays. Recent abundance estimates (2011 and 2012) however, are lower than all previous estimates (1979–1981, 1989–1992 and 2003), indicating a possible long-term impact. Quantification of the spatial and temporal partitioning of wildlife behavioural schedules provides critical insight for conservation measures that aim to mitigate the effects of human disturbance.

## 1. Introduction

To facilitate survival, animals assess the risks associated with selecting appropriate habitats for important behavioural activities [1]. The selective forces associated with choosing one habitat over another shape the evolution of behavioural strategies [2], and influence the temporal and spatial partitioning of behavioural activities [3].

Behavioural strategies can be considered flexible or constrained. For example, the bushbuck (*Tragelaphus scriptus*), a medium sized antelope [4], the African elephant (*Loxodonta africana*) [5] and the bottlenose dolphin (*Tursiops truncatus*) [6] readily alternate between behavioural states throughout their day, often within the same habitat, indicating flexible behavioural strategies. By contrast, spinner dolphins (*Stenella longirostris*) in Hawaii have evolved a predictable diel behavioural strategy that is spatially and temporally constrained.

In the 1970s, pioneering work by Norris *et al*. [7,8] described the diel behavioural activities of the spinner dolphins of Hawaii. They showed that spinner dolphins forage offshore at night, returning to preferred sheltered coastal areas to socialize and rest during the day [7,8]. From this work, they proposed two hypotheses that have since been documented: (i) spinner dolphins feed cooperatively on prey of the mesopelagic boundary community [9]; and (ii) certain bays are used for resting, apart from reducing predation risk, because of their proximity to deep water foraging grounds [10]. Norris *et al*. [7] showed that on arrival at their resting bays spinner dolphins initially socialize before descending into rest, typically between 10.00 and midday. When they awaken, they socialize before travelling out to their night-time foraging grounds [7,8]. More recently, a study documented that spinner dolphins are unlikely to rest outside of these bays [11]. Consequently, important behaviours (foraging and resting) are spatially and temporally segregated. This behavioural strategy allows spinner dolphins to maximize their foraging efficiency while minimizing predation risk during vulnerable resting periods [12].

Increasingly, free-ranging animals are exposed to anthropogenic activities [13], which introduces additional pressures affecting their health and fitness. Perturbation through anthropogenic activities can affect the health of individuals through lost time conducting important behaviours (e.g. foraging and resting), leading to negative impacts on vital rates and population viability [13]. To compensate for disturbance, animals can either move away from the source of disturbance to continue their current activity elsewhere or move away and then return to the location of disturbance once it has passed and resume their prior activity.

A measure of a system's ability to compensate and persist in the face of disturbance is defined as *resilience* [14]. Resilience theory suggests that systems which are constrained in their behavioural repertoire are less resilient to disturbance than flexible systems [15]. It therefore follows that species with constrained and predictable behavioural patterns may be less able to compensate for disturbance, compared with species with more variable behavioural patterns.

Using a combination of land- and boat-based behavioural time-series observations, we investigated the temporal and spatial partitioning of behavioural activities of Hawaii Island spinner dolphins. This population is exposed to high levels of anthropogenic disturbance in the form of wildlife tourism [16], which has the potential to threaten important behaviours such as resting. Hence understanding the behavioural plasticity of this species will help determine its resilience to anthropogenic disturbance.

## 2. Methods

### 2.1. Fieldwork

**Figure 1.**
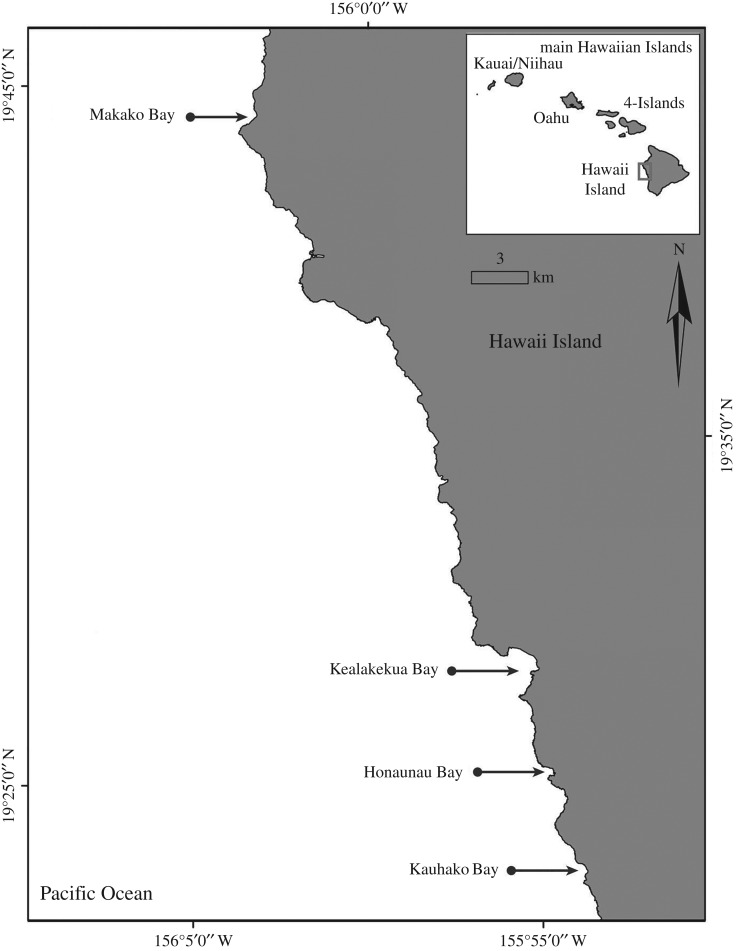
Map of the study area illustrating the four spinner dolphin resting bays, Makako Bay, Kealakekua Bay, Honaunau Bay and Kauhako Bay, along the Kona Coast of Hawaii Island.

Between September 2010 and December 2012, land-based and boat-based sampling protocols were used to document spinner dolphin behaviours in coastal waters off the Kona Coast, on the leeward side of Hawaii Island. Specifically, dolphin behaviours were recorded via boat-based surveys inside and outside (within 1 km of the coastline) of four sheltered bays: Makako Bay, Kealakekua Bay, Honaunau Bay and Kauhako Bay (figure 1). In addition, land-based group focal follows were undertaken via theodolite tracking from clifftops overlooking Kauhako Bay and Kealakekua Bay.

### 2.2. Group focal follows

Established group focal follow protocols were employed to collect positional and behavioural time-series information on spinner dolphins during daylight hours from both the boat- and land-based platforms. Group focal follows consisted of a combination of continuous and instantaneous scan sampling procedures [17]. Instantaneous scan sampling was used to record the predominant group activity of the dolphins (table 1) at 10 min intervals. Group focal follows were terminated when behaviours could no longer be reliably determined owing to either poor visibility, dolphins moving out of range or splitting into too many groups. Further details of focal follow protocols are given in [11].

**Table 1.** Definitions of spinner dolphin group behavioural activities, adapted from Norris *et al.* [8].

predominant group behavioural activity	
resting	characterized by tight grouping, slow swim speed with dolphins moving back and forth or meandering. Individuals typically take multiple breaths within a surfacing bout. Synchronous group diving; spending long periods of time submerged (1.5–3 min)
socializing	characterized by regular, consistent, aerial behaviours within the group; little time spent below the surface; brief dives
travelling	characterized by regular and consistent movement, i.e. directed swimming that was roughly straight. Travel speed was typically 3.2 km h^−1^
foraging	characterized by asynchronous dives of large widely dispersed groups. Pre-dive, groups are evident with much aerial behaviour across groups; the dives may start at dusk; groups dive individually following within a minute or two; dives are long averaging 3.5 min

### 2.3. Data analysis

To quantitatively describe the behaviour of spinner dolphins, boat-based and land-based behavioural data were combined to determine the probability of resting, socializing and travelling as a function of time-of-day (hour). Generalized additive mixed models (GAMM; gamm in R package mgcv) were used with a thin plate regression spline smoother and a binomial distribution and logit link function. A separate model was fitted to each behavioural activity. As sequential observations within focal follows could not be considered independent, a temporal auto-correlation structure within follows was incorporated into the model, where the residuals at any given time were modelled as a function of the residuals of the previous time points. The most suitable auto-correlation structure was fitted by altering the number of auto-regressive and moving average parameters and then comparing the different models. Auto-correlation and partial auto-correlation function plots were used to detect patterns of auto-regressive and moving average parameters visually, before and after adding the different correlation structures. All three models included an auto-correlation within focal follows, with a lag of one (10 min).

## 3. Results

### 3.1. Behavioural sampling effort

A total of 105 boat- and land-based dolphin group focal follows were conducted resulting in 428 h of focal follow data (table 2).

**Table 2.** Number and duration of focal follows collected from land- and boat-based platforms inside and outside of resting bays along the Kona Coast, Hawaii Island.

platform and location	no. focal follows	focal follow effort (h)	mean focal follow duration (hh.mm ± s.e.)
land-based			
Kealakekua Bay	23	189	08.27 ± 00.19
Kauhako Bay	7	38	03.25 ± 01.17
total	30	227	07.57 ± 00.22
boat-based			
Makako Bay	13	26	02.00 ± 00.26
Kealakekua Bay	10	16	01.36 ± 00.15
Honaunau Bay	5	21	04.12 ± 00.15
Kauhako Bay	10	16	01.36 ± 00.48
outside bays	37	117	03.10 ± 00.13
total	75	201	02.41 ± 00.10
**overall total**	**105**	**428**	**04.08 ± 00.51**

### 3.2. Partitioning of behaviours

**Figure 2.**
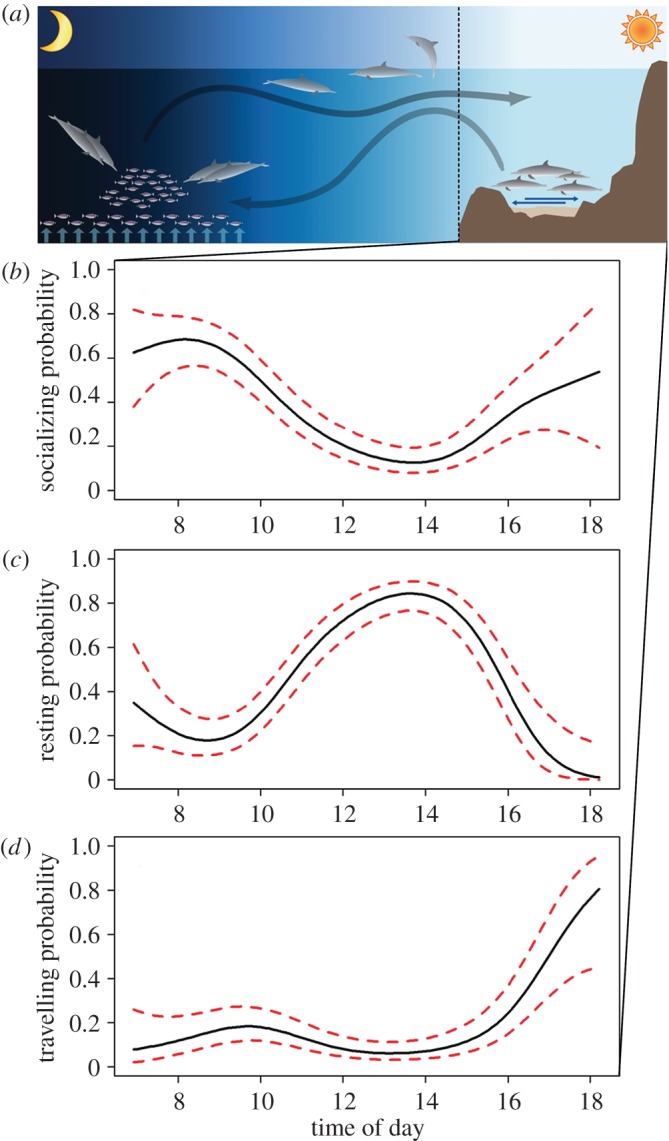
Schematic diagram of the diel behavioural pattern of spinner dolphins (*a*), adapted from Johnston [12]. Diurnal probability of (*b*) socializing, (*c*) resting and (*d*) travelling as a function of time-of-day, estimated from the 428 h of focal follow observations. The solid black lines represent the fitted values of the GAMMs and the dashed red lines represent the 95% CIs.

Based on our land-based theodolite observations (227 h), we documented that after a night of foraging, spinner dolphins returned to sheltered bays to socialize, rest and avoid predators on a daily basis (figure 2*a*). Specifically, dolphins entered Kealakekua Bay and Kauhako Bay at 07.28 (s.d. ± 28 min), and exited again at 16.53 (s.d. ± 41 min); dolphins were never observed foraging.

During daytime, an inverse bell-shaped relationship was found between the probability of socializing (*F*_5.0,2507.0 _= 11.7, *p* < 0.001, deviance explained = 18.7%) and time-of-day, with peaks in activity occurring between 07.00 and 09.00, and between 16.00 and 18.00, separated by a low level of socializing between 12.00 and 15.00 (figure 2*b*). By contrast, we documented a bell-shaped relationship between the probability of resting and time-of-day (*F*_5.6,2506.4 _= 16.9, *p* < 0.001, deviance explained = 25.7%), with a peak in resting activity between 12.00 and 14.00 and a lower probability of resting during early morning and late afternoon (figure 2*c*). An inverse curvilinear relationship was found between the probability of travelling (*F*_4.5,2507.5 _= 7.1, *p* < 0.001, deviance explained = 2.7%) and time-of-day with a smaller peak in activity between 09.00 and 11.00, followed by a low level of activity between 12.00 and 15.00, which was followed by a rapid increase in activity after 16.00 (figure 2*d*). The dispersion parameters (*φ*) for the socializing, resting and travelling models were 0.97, 0.96 and 0.79, respectively, which indicated no over-dispersion of the binomial GAMMs.

## 4. Discussion

The temporally and spatially partitioned behavioural activities and habitat selection strategies employed by island-associated spinner dolphins have evolved into a stable strategy that constrains their diel behavioural repertoire. Data presented here corroborate Norris *et al*.'s earlier work (from the late 1970s to the early 1980s). It was at this time that Norris *et al.* presented a cautionary tale: ‘Kealakekua's waters are a reserve now, but many boats continue to use the bay…and if their number increases, if the dolphins' needs aren't considered, the animals will leave and their span of tenancy, which began before that of any man will end as they quietly slip away into the offshore sea’. [18, p. 67]. Even though spinner dolphins still inhabit these bays after more than 30 years, the cetacean tourism industry has grown significantly in Hawaii in the past 10–15 years [19]. The most recent abundance estimates from 2011 [20] and 2012 [21] are lower than all previous estimates 1979–1981 [8], 1989–1992 [22] and 2003 [23], indicating a possible long-term population level impact.

Spinner dolphins forage at night on prey that migrates vertically towards the ocean surface from the mesopelagic layer [7–9]. We show that dolphins mainly rest between 10.00 and 16.00 upon their return to sheltered near shore habitats. Resting in dolphins is the most sensitive activity to interactions with humans [24]. Consequently, human disturbance to resting behaviour will have the greatest impact on their daytime behavioural budget [25]. We also document that socializing behaviour occurred in the early mornings and late afternoons within bays. Reinforcing social bonds and social cohesion between conspecifics may be important for successful cooperative night-time foraging [26]. We are not aware of any other cetacean species that partitions its behavioural activities in such a temporally and spatially constrained manner on a 24 h basis. Following the theory of resilience of natural systems [14], such constraints may render spinner dolphins less able to compensate for disruptions to their behavioural schedule. Consequently, it is likely that they are more vulnerable to disturbance which, in turn, can lead to long-term population impacts.

This small [20,21], genetically isolated [27], spinner dolphin population is chronically exposed to human tourism activities for more than 82% of the time during daytime hours with a median interval between exposure events of only 10 min [16]. To our knowledge, these are the highest reported exposure rates of any free-ranging coastal dolphins to targeted tourism activities.

Habitats chosen by spinners dolphins during the daytime are important because they facilitate survival by reducing the risk of predation during vulnerable resting periods [7,8] and are proximal to the deep water foraging grounds [7,8,10]. Should the continued exposure to human activities cause dolphins to abandon preferred daytime habitats, they will probably be more vulnerable to predation and be more vigilant (and hence engage in less resting behaviour) which may cause population-level effects. While there are caveats with comparing with previous estimates [20], there are early indications of possible detrimental long-term impacts: two recent consecutive abundance estimates of 631 (95% confidence interval (CI): 524–761; [20]) and 668 (95% CI: 556–801; [21]) individuals are lower than any previous published estimates, 960 [8], 2334 [22] and 855–1001 individuals [23].

Understanding the behavioural schedule of spinner dolphins is of critical importance for the development of conservation measures that effectively mitigate their exposure to human activities and assist in their resilience in the face of disturbance. This is especially important in the main Hawaiian Islands where the dolphins are chronically exposed to tourism in their important resting habitat, where vessels closely approach them and people enter the water to interact with them while the dolphins are trying to rest. Island- and atoll associated spinner dolphins elsewhere (e.g. Samadai Reef, Egypt, Red Sea [28], Fernando de Noronha Archipelago, Brazil [29], Moon Reef, Fiji [30]) also exhibit similar diel behavioural strategies, suggesting they may also be less resilient and therefore more vulnerable to disturbance.

## Data accessibility

The datasets supporting this article are available from the Dryad data repository: http://dx.doi.org/10.5061/dryad.td065 [31].
